# The Book World of Medicine and Science

**Published:** 1900-01-20

**Authors:** 


### The Book World of Medicine and Science.

On the Prevention of Eye Accidents Occurring in Trades. By Simeon Snell, F.R.C.S.Edin. (London: J. Bale, Sons, and Danielsson. 1899. 32pp. Plates. Price 1s.)

Mr. Snell's address to the section of ophthalmology at the meeting of the British Medical Association will be welcome in its present form, not only to the profession, but, perhaps, also to those interested in the improvement of industrial conditions. The risks to eyesight in workmen employed in the chipping of iron or steel castings are shown by the author to be as numerous as they are readily prevented, and it is impossible to doubt that, were his excellent and practical suggestions carried out, such accidents would be very largely reduced in number. Unfortunately, as is so often the case, the opposition to preventive measures comes from the men themselves, who object to wearing gauze protectors while at work, though the exemption secured where their use is compulsory will doubtless lessen the prejudice against them. The author also draws attention to the large number of foreign bodies removed from the cornea by some workmen who enjoy a reputation for dexterity at this operation. The skill of the operator, so far as it goes, forms a poor compensation for the rough and septic instruments employed. Lancets, pocket-knives, and pins, frequently wetted with saliva, are not calculated to ensure success even in most careful hands. As Mr. Snell points out, it is probable that the Workmen's Compensation Act will tend to greater care in the prevention of such accidents.

British Sanatoria for the Open-air Treatment of Tuberculosis. (London: J. Bale, Sons, and Danielsson. Pp. 50. Illustrated. Price 1s. 6d. 1899.)

This useful guide to the sanatoria for the open-air treatment of phthisis in Great Britain has been reprinted from the *West London Medical Journal*, and will doubtless enjoy a larger circulation in its present form. It contains an alphabetical list of the institutions, with much useful information as to their sites, surroundings, and the systems of treatment therein practised, illustrated in many cases by sketches and photographs of the houses and grounds. Here, likewise, the practitioner may find information as to the dietary, accommodation, and charges, as well as many other details which may assist him in selecting a resort for the particular patient whose case he has in view. The book is evidence of the great and increasing interest in the treatment of consumption by residence in the open air, and though much can be done in this way at home when the surroundings of the patient are such that it can be carried out, yet the regular life, diet, and exercise of a special institution are undoubtedly much more efficacious. To those intending undergoing such a period of residence, and to their medical attendants, we have much pleasure in commending this guide.

The Medical Digest, or Busy Practitioner's Vade-mecum. Appendix, including the years 1891 to March, 1899. By Richard Neale, M.D.Lond. (London: John Bale, Sons, and Danielsson. 1899. Price 15s. 6d. net.)

Everyone knows "Neale's Digest," and everyone who has used it is bound to confess that without it he would find it impossible to find his way through the voluminous utterances of bygone medical journalism. Naturally such a work requires to be brought up to date as years go by, so that the occasional issue of an appendix becomes necessary, and as in this appendix the same general plan is followed as that on which the original work was constructed, all the praise which "Neale's Digest" has won for itself may properly be held to apply also to the work now before us—if we except one point. With what we cannot but regard as extraordinary short-sightedness this book has been so arranged that it can only be used as an appendix to the older volume. By itself it is useless, for it is unindexed. To use the appendix "the Index of Edition of 1890 must first be consulted for any subject, and the corresponding section referred to in the Appendix to see if fresh matter has been added. If the index of 1890 does not refer to the subject sought for then turn to Appendix Index, in which new subjects are alone noted." Hence the book is useless to all except those who possess the old volume, and even they must look through two indices before they can ascertain whether or not a subject is referred to. And yet what a handy book it would have been if it had had a proper index! We have seldom seen such an example of the spoiling of a good ship for the sake of a bucketful of tar.

Dictionnaire de Physiologie. Par Charles Richet. Deuxième Fascicule de Tome IV. (Paris: Félix Alcau. 1899.)

The cyclopædic enterprise of "fin de siècle" authors shows no sign of abatement. Contemporaneously with the enormous series of volumes on medicine, surgery, and physiology now issuing from English and American presses we have this monumental work, of which the second fasciculus of the fourth volume only carries us from Cœur to Cyanhydisque! And this in three hundred closely-printed, large quarto pages. It is true that "physiology," as understood by Richet, is "physiology" of an even ampler scope than the physiology of our medical schools. It is physiology in the widest sense, and in this dictionary space is found for monographs on Consanguinity, on Crustaceans, and on Crotonic Acid. The generosity of treatment allowed may be inferred when we say that to Creatin alone are devoted six or seven quarto pages of small print. Such an undertaking as this reminds us of Macaulay's criticism of Dr. Laws' work, of which the volumes were each said to rival a library, the preface a book, and the chapters separate treatises. The writers whose work M. Richet co-ordinates are French, Russian, German, and Italian authors of European celebrity. But no Englishman, we notice, contributes, and references to English authors are very few, even though America is freely drawn upon. Such narrowness detracts not a little from the practical value of the work to Frenchmen, but we freely acknowledge the admirable lucidity, directness, and unfailing logic of those of the monographs which are from Gallic pens. Some of the articles, those on Consanguinity and on the Cornea, for example, are almost perfect examples of that happy combination of literary purity and scientific clarity in which the countrymen of Descartes and Pascal excel. But in fact the work bids fair to be, when complete, a library of reference, not only for the physiologist, but for the physician, zoologist, botanist, and pharmacist alike.

Pocket Notes on the British Pharmacopœia. (1898.)

Under the above title we have had sent to us a neat little book for the pocket which presents in a convenient and attractive form the chief alterations and additions to the "British Pharmacopœia" made in 1898. This little booklet is issued by Messrs. Ferris and Co., the well-known firm of Bristol druggists.

